# Probiotic Effect on Quality of Life, Bowel Habits and Weight Loss after Different Types of Bariatric Surgeries, a Randomized Controlled Trial

**DOI:** 10.1007/s11695-026-08706-1

**Published:** 2026-05-06

**Authors:** Mostafa Mahmoud Abdelfatah, Osama Salah Mohammad, Ismail Ahmed Shafik, Mahmoud Ali Mohammed AbdelMohsen

**Affiliations:** Kasralainy School of Medicine, Cairo University, Giza, Egypt

**Keywords:** Probiotic effect, Quality of life, Bowel habits, Weight loss, Bariatric surgeries

## Abstract

**Introduction:**

Obesity is a complex health condition marked by an excessive accumulation of adipose tissue. Bariatric surgery continues to be the most efficacious intervention for attaining significant and enduring weight loss. This study aimed to assess a 6-month probiotics supplementation effect on quality of life (QoL), bowel habits, excess weight loss (EWL) and changes in stool composition and fecal Calprotectin in severely obese patients undergoing different types of bariatric surgeries.

**Methods:**

This prospective randomized controlled trial involved 200 cases underwent standardized bariatric procedures. Cases were randomized into two equal groups: Probiotic group received either Enterogermina^®^ 6 billion sachets or Lacteol forte^®^ sachets, one sachet twice daily for 6 months postoperatively and control group: Patients received standard postoperative care without probiotics.

**Results:**

At 1 month postoperatively, the probiotics group showed a significantly higher QOL and EWL% (*p* < 0.001) compared to controls. At 3 months, the probiotics group had a significantly higher QOL and EWL% (*p* < 0.001). At 6 months, the probiotics group showed a statistically significant improvements across all measured outcomes, including QOL, BHQ, and EWL% compared to the control group(*p* < 0.001). Probiotic group had a significantly lower fecal calprotectin compared to control group (*p* < 0.001).

**Conclusion:**

The probiotic use post-bariatric surgery offers measurable benefits in QOL, weight loss, and gastrointestinal inflammation, regardless of the surgical type. Although procedures like RYGB and OAGB yield superior weight loss, they may induce higher gut inflammation. Meanwhile, sleeve gastrectomy appears to be the most gut-friendly in terms of inflammatory markers and bowel consistency.

## Key points

We evaluate the impact of six months of probiotic supplementation on quality of life (QoL), bowel habits, excess weight loss (EWL), and alterations in stool composition and fecal Calprotectin levels in severely obese cases undergoing various forms of bariatric surgery.

Patients who underwent OAGB and RYGB had significantly higher EWL than those who had sleeve gastrectomy and gastric plication.

Probiotics had significantly lower fecal calprotectin (*p* = 0.000). Also, there stool analysis was significantly different between both groups.

Probiotic use post-bariatric surgery offers measurable benefits in QoL, weight loss, and gastrointestinal inflammation, regardless of the surgical type.

## Introduction

Obesity is a complex health condition characterized by excessive fat accumulation, which greatly raises the risk of both physical and mental health problems, including type 2 diabetes, hypertension, and cardiovascular disease [1]. Bariatric surgery continues to be effective intervention for attaining significant weight loss in individuals with severe obesity [2]. The effects of specific bariatric procedures on bowel habit are not well defined. Some reports have suggested that the Roux-en-Y gastric bypass (RYGB) and biliopancreatic diversion (BPD) result in diarrhea. In contrast, others have reported improvement in symptoms of loose stools after RYGB. Most reports associate the adjustable gastric band (AGB) and sleeve gastrectomy (SG) with constipation [3]. Several patients have reported experiencing episodes of gastrointestinal symptoms following bariatric surgery, and these symptoms are correlated with diminished quality of life (QoL) [4].

Probiotics, defined as live microorganisms that confer health benefits when administered in adequate amounts, have been proposed as potential adjuncts in managing obesity [5]. The concept of using probiotics to support post-bariatric surgery patients is rooted in their potential to impact several physiological processes. Probiotics may improve gut health, and modulate inflammatory responses, which can benefit individuals recovering from bariatric surgery [6].

Furthermore, evidence suggests that probiotics could influence appetite regulation and energy metabolism, which are critical factors in weight management and obesity treatment [7]. However, the use of probiotics in this context remains underexplored, particularly given the heterogeneous nature of post-bariatric surgery patients. Factors such as the type of surgical procedure, individual variations in gut microbiota, and differing probiotic strains could all impact the effectiveness of these supplements. This variability underscores the need for a comprehensive evaluation of how probiotics might benefit post-bariatric surgery patients and whether they can reliably contribute to sustained weight loss and improved metabolic health [8]. Research indicates that individuals with obesity frequently exhibit reduced microbial diversity and an elevated Firmicutes-to-Bacteroidetes ratio in comparison to slender individuals [9].

The aim of our study was to evaluate the effect of a 6-month probiotics supplementation on quality of life, bowel habits, excess weight loss and changes in stool composition and fecal Calprotectin in 100 severly obese patients undergoing different types of bariatric surgeries.

## Methods

This prospective randomized controlled trial involved 200 cases underwent standardized bariatric procedures at the General Surgery Department of Kasr ElAiny Hospital, Cairo University between May 2024 and August 2025. The study was designed to evaluate probiotics effect on bowel habits, stool composition, and related outcomes after various bariatric surgeries.

Inclusion criteria were any patient both genders, aged 18 to 65 years, BMI > 35 kg/m² with or without comorbidities or body mass index (BMI) > 30 kg/m² with comorbidities, scheduled for any bariatric surgery: laparoscopic SG (LSG), laparoscopic greater curvature plication, laparoscopic one anastomosis gastric bypass, or laparoscopic RYGB (LRYGB). The selection of the bariatric surgical procedure was determined through mutual consensus between the patient and the surgeon.

Exclusion criteria were patients on antibiotic use within 30 days prior to surgery, probiotic use within 30 days prior to surgery, patients using proton pump inhibitors (PPIs) or potassium-competitive acid blockers (P-CABs), gastrointestinal infections or inflammatory bowel disease, thyroid disorders, history of cancer, particularly of the digestive tract and immunodeficiency.

The type of bariatric surgery was decided by mutual consensus between the patient and the surgeon.

All patients were subjected to the following demographic data (sex, age, residence), medical history, anthropometrics measurements (anthropometrics: weight and BMI at each visit to calculate % EWL), type of bariatric surgery, and comorbidities. All patients were administered standard pre-operative prophylactic antibiotics to mitigate the risk of surgical site infection.

### Randomization and Blindness

Patients were then randomly allocated by opening sequentially numbered opaque sealed envelopes. The allocation sequence was generated using a computer-generated random number list with an allocation ratio of 1:1. Patients were randomized into two equal groups:

- **Probiotic group (n = 100)**: Cases received either Enterogermina^®^ 6 billion sachets (Bacillus clausii spores) or Lacteol forte^®^ sachets (Lactobacillus LB, 10 billion, lyophilized inactive microbial bodies), one sachet twice daily for 6 months postoperatively. Compliance was monitored via sachet count and monthly visits.
- **Control group (n = 100)**: Patients received standard postoperative care without probiotics.

Both participants and outcome assessor were blinded to the allocation group.

#### Laboratory tests

At 3 and 6 months: CBC, iron, transferrin, total and ionized calcium, parathormone, urea, creatinine, magnesium, zinc, fasting glucose, vitamin B12, vitamin D, lipid profile, AST, ALT, HbA1c.

#### Stool analysis & calprotectin

Collected at baseline and 6 months.

#### Surgical outcomes

Operative time, intraoperative findings, hospital stay, complications.

### Bowel Habits & QoL

Health-related quality of life was evaluated utilizing the 36-Item Short Form Health Survey (SF-36). The questionnaire assesses eight aspects of health status: physical functioning, physiological discomfort, role limitations attributable to physical health, social functioning, overall health perceptions, vitality, role limitations resulting from emotional problems, and mental health. The SF36 comprises 36 questions and typically takes approximately 8 to 10 min to complete independently. Responses are systematically coded and converted into a 0–100 scale, with higher scores representing greater health. Domain scores are determined by aggregating the item values across each of the eight dimensions [10]. Table 1.

**Table 1:** Interpretation of CDC bowel health questionnaire scores

Measure/Domain	Score Range (or Categories)	Interpretation
Fecal incontinence severity index (FISI)	Weighted 1–20 per symptom; Total FISI = sum of weights	Higher scores indicate more severe symptoms of fecal incontinence involving gas, mucus, liquid, and solid stool.
Frequency of bowel movements	Numeric: times per week (converted from per day/week responses)	Indicates usual defecation frequency. Useful to categorize: constipated (< 3/week), normal (3–21/week), or high frequency (> 21/week).
Stool consistency (bristol stool scale)	1–7 scale: 1 = separate hard lumps (constipation) 4 = smooth, soft (ideal) 7 = entirely liquid (diarrhoea)	Lower scores (1–2) reflect constipation; midrange (3–5) indicate normal; higher scores (6–7) suggest diarrhoea or faster transit. Ideal stool = Type 4.

Bowel health was evaluated using the Bowel Health Questionnaire (BHQ) [11]. The BHQ is a validated, self-administered tool designed to assess stool frequency, stool consistency, bowel leakage, use of laxatives or stool softeners, and the presence of blood in the stool. Unlike multidimensional health surveys such as the SF36, the BHQ does not produce an overall summary score; instead, outcomes are analysed on an item-by-item basis and expressed as categorical distributions and prevalence rates across the study population. Table 1.

### Outcomes

Primary outcome: Effect of probiotic use on bowel health and bowel habits after bariatric surgery.

Secondary outcomes included the effect of probiotic supplementation and QoL (SF36, MA QoL QII) and %EWL, fecal calprotectin, stool analysis, and culture, relationship between stool composition and maldigestion and incidence of gastrointestinal symptoms postoperatively and complications.

Follow up was scheduled at 1, 3, and 6 months postoperatively alongside the standard post bariatric protocol. Compliance with probiotics was assessed at each visit.

### Statistical Analysis

Statistical analysis was performed by SPSS version 25 (IBM Inc., ARMONK, NY, USA). Shapiro-Wilks normality test and histograms were used to test the distribution of quantitative variables to select accordingly the type of statistical testing: parametric or nonparametric. Parametric variables were expressed as mean and standard deviation (SD) and were compared using ANOVA test among the three groups with post hoc (Tukey) test to compare groups. Repeated measures ANOVA tests was used to analyze differences across multiple time points or conditions with the same participants. Categorial variables were expressed as frequency and percentage and were statistically analyzed by Chi-square test. A two-tailed P value ≤ 0.05 was considered statistically significant.

## Results

There was an insignificant difference between both groups regarding age, gender, type of surgery, and associated comorbidities as DM or HTN. Preoperatively, there was an insignificant difference between probiotic and control groups regarding BMI, QOL, BHQ, and fecal calprotectin. Table 2.

**Table 2:** Basic demographic characteristics and preoperative data of all patients (*n* = 200)

**P**	**Control (n=100)**	Probiotics (n=100)	Total (n=200)		
0.102	**Gender**				
	40 (40%)	29 (29%)	69 (34.5%)	**Male**	
	60 (60%)	71 (71%)	131 (65.5%)	**Female**	
0.071	37.1±9.6	35.25±9.11	36.98±9.5	**Age in years**	
0.998	**Type of surgery**				
	43 (43%)	44 (44%)	87 (43.5%)	**Sleeve gastrectomy**	
	19 (19%)	19 (19%)	38 (19%)	**Gastric plication**	
	19 (19%)	19 (19%)	38 (19%)	**OAGB**	
	19 (19%)	18 (18%)	37 (18.5%)	**RYGB**	
**Preoperative data**					
0.295^a^	42.87±5.67	42.61±6.48	--	**BMI**	
0.254^a^	82.47±5.9	83.53±3.9	--	**QOL**	
0.931^a^	82.69±4.6	82.66±5.2	--	**BHQ**	
1.00	50 (50%)	50 (50%)	--	**Formed**	**Stool analysis**
	50 (50%)	50 (50%)	--	**loose**	
--	100 (100%)	100 (100%)	--	**Normal GIT flora**	**Stool culture**
0.696^a^	163.52±53.6	167.19±46.77		**Fecal calprotectin**	

Data is presented as mean ± SD or frequency (%), *QOL* quality of life, *BHQ* Bowel Health Questionnaire, *EWL* Early postoperative weight loss, *OAGB* one Anastomosis Gastric Bypass, *BMI* body mass index. *RYGB* Roux-en-Y gastric bypass, *a* Chi square test, *b* Mann Whitney test, *QOL* quality of life, *BHQ* bowel habit questionnaire

Table 3 demonstrates that, within the probiotics group, both QOL and BHQ scores remained statistically insignificant across follow-up intervals, whereas EWL% showed a significant increase as EWL% was significantly higher at 3 and 6 months compared to 1 month (*P* < 0.001 for both), and further improved at 6 months relative to 3 months (*P* < 0.001). In the control group, BHQ scores were statistically insignificant across different time points, while QOL was significantly higher at 6 months compared to 1 month (*P* = 0.011), with no other statistically significant differences observed. Similarly, EWL% in the control group increased significantly at 3 and 6 months compared to 1 month (*P* < 0.001 for both), and was enhanced at 6 months compared to 3 months (*P* < 0.001). Between-group comparisons revealed that, at 1 month postoperatively, the probiotics group showed a significantly higher QOL and EWL% (*p* < 0.001) compared to controls, with no significant difference regarding BHQ. At 3 months, the probiotics group had a significantly higher QOL and EWL% (*p* < 0.001), while BHQ remained statistically insignificant between both groups. At 6 months, the probiotics group showed a statistically significant improvements across all measured outcomes, including QOL, BHQ, and EWL% compared to the control group(*p* < 0.001). Stool analysis was significantly different between both groups. Probiotic group had a significantly lower fecal calprotectin compared to control group (*p* < 0.001). Table 4.

**Table 3:** Postoperative data after 1 month, 3 months and 6 months within the studied groups

		1 month	3 months	6 months	P	
QOL	**Probiotics (n = 100)**	86.38 ± 4.32	87.03 ± 3.9	87.25 ± 3.8	0.282	
	**Controls (n = 100)**	83.2 ± 4.5	84.07 ± 4.5	84.84 ± 4.5	0.037*	P1 = 0.173, P2 = 0.011*, P3 = 0.228
	**P value between group**	< 0.001*	< 0.001*	< 0.001*	--	
BHQ	**Probiotics (n = 100)**	83.22 ± 4.9	83.3 ± 4.7	84.20 ± 4.26	0.252	
	**Controls (n = 100)**	81.83 ± 4.9	82.43 ± 4.9	82.74 ± 4.44	0.389	
	**P value between group**	0.054	0.237			
EWL %	**Probiotics (n = 100)**	9.44 ± 2.33	29.6 ± 4.7	57.47 ± 7.8	< 0.001*	P1 < 0.001*, P2 < 0.001*, P3 < 0.001*
	**Controls (n = 100)**	7.54 ± 1.98	24.6 ± 4.7	42.36 ± 5.7	< 0.001*	P1 < 0.001*, P2 < 0.001*, P3 < 0.001*
	**P value between group**	< 0.001*	< 0.001*	< 0.001*		

Data is presented as mean ± SD or frequency (%), *QOL* quality of life, *BHQ* Bowel Health Questionnaire, *EWL* Early postoperative weight loss, *: statistically significant as p value < 0.05, P1: p value between 1 and 3 months, P2: p value between 1 and 6 months, P3: p value between 3 and 6 months

**Table 4:** Stool analysis fecal calprotectin at 6 months

		Probiotics (*n* = 100)	Controls (*n* = 100)	*P* *
Stool analysis	Formed	41 (41%)	61 (61%)	0.005 ^b^
	loose	59 (59%)	39 (39%)	
Fecal calprotectin		88.56 ± 33.8	138.64 ± 35.03	**< 0.001** ^**a**^

Data is presented as mean ± SD or frequency (%), *: Mann Whitney test. b: Chi square test

Upon comparing baseline and 6 months postoperative parameters, QOL, EWL% showed a significantly increased in both the probiotics group and the control group (*p* < 0.001), and the probiotics group had a significantly higher percentage than controls (*p* = 0.049, 0.014 respectively). Fecal calprotectin levels significantly decreased in both the probiotics group and the control group (*p* < 0.001), with the probiotics group exhibiting a significantly greater percentage reduction than controls (*p* < 0.001). BHQ scores had a significantly increased in the probiotics group (*p* = 0.001), while was insignificantly different in the control group. Table 5.

**Table 5:** Pre/postoperative change among patients

	Probiotics (*n* = 100)			Controls (*n* = 100)		
	Baseline	6 months	*P* *	Baseline	6 months	*P* *
QOL	83.53 ± 3.9	87.25 ± 3.8	**< 0.001**	82.47 ± 5.9	84.84 ± 4.5	**< 0.001**
BHQ	82.66 ± 5.2	84.20 ± 4.26	**< 0.001**	82.69 ± 4.6	82.74 ± 4.44	0.913
EWL %	9.44 ± 2.33	57.47 ± 7.8	**< 0.001**	7.54 ± 1.98	42.36 ± 5.7	**< 0.001**
Fecal calprotectin	167.19 ± 46.77	88.56 ± 33.8	**< 0.001**	163.52 ± 53.6	138.64 ± 35.03	**< 0.001**
Postoperative percent change						P *
QOL	3.57% (1.2:6.9)			3.47% (0.0:5.3)		**0.049**
BHQ	2.24% (−1.16:5.05)			−0.57% (−2.5:2.6)		**< 0.001**
EWL % †	509.5% (459.6:583.3)			484.5% (392.5:570.2)		**0.014**
Fecal calprotectin	−48.9% (−64.04: −23.2)			−7.44% (−33.8:8.7)		**< 0.001**

Data is presented as mean ± SD or frequency (%), *QOL* quality of life, *BHQ* Bowel Health Questionnaire, *EWL* Early postoperative weight loss, *: Paired samples T test. *: Mann Whitney test. †: EWL from 1 m till 6 m postoperative

Regarding postoperative change of stool analysis, probiotics group showed more loose stool motions without significant difference between groups. Table 6.

**Table 6:** Postoperative change of stool analysis among patients

		Baseline		6 months		*P* *
		Probiotics	Controls	Probiotics	Controls	
Stool analysis	**Formed**	50 (50%)	50 (50%)	41 (41%)	61 (61%)	0.915
	**loose**	50 (50%)	50 (50%)	59 (59%)	39 (39%)	
Stool culture	**Normal GIT flora**	100 (100%)	100 (100%)	100 (100%)	100 (100%)	-

Data is presented as mean ± SD or frequency (%), *: Mc Nemar test

According to the type of bariatric surgery among patients who received probiotics, preoperative assessments revealed a statistically insignificant differences between surgical groups as regard BMI, QOL, BHQ, and fecal calprotectin. At 1 month postoperatively, patients who underwent RYGB demonstrated a significantly higher EWL% compared to those who underwent SG, gastric plication, or OAGB, while there was no statistically significant difference regarding QOL or BHQ. At 3 months, RYGB patients showed a significantly higher EWL% compared to SG and gastric plication, while QOL and BHQ remaining statistically insignificant among the groups. At 6 months, RYGB patients exhibited a significantly higher EWL% compared to SG and gastric plication. Additionally, SG patients had a significantly lower fecal calprotectin levels compared to those who underwent OAGB and RYGB, while QOL and BHQ differences remained a statistically insignificant. Table 7.

**Table 7:** Preoperative data and postoperative data after 1 month, 3 months and 6 months

		Sleeve gastrectomy (*n* = 44)	Gastric plication (*n* = 19)	OAGB (*n* = 19)	RYGB (*n* = 18)	*P*	Post hoc analysis
Preoperative data							
**BMI**		42.98 ± 6.4	41.58 ± 6.8	41.05 ± 6.5	44.4 ± 6.2	0.372 ^a^	--
**QOL**		83.41 ± 4.3	84.2 ± 3.9	84.21 ± 3.1	82.4 ± 3.7	0.443 ^a^	--
**BHQ**		82.27 ± 5.2	84.05 ± 5.4	82.63 ± 5.44	82.2 ± 5.07	0.631 ^a^	--
**Stool analysis**	**Formed**	24 (54.5%)	8 (42.1%)	10 (52.6%)	8 (44.4%)	0.774 ^b^	--
	**loose**	20 (45.5%)	11 (57.9%)	9 (47.4%)	10 (55.6%)		
**Stool culture**	**Normal GIT flora**	44 (100%)	19 (100%)	19 (100%)	18 (100%)	-	--
**Fecal calprotectin**		166.07 ± 45.9	163.6 ± 52.5	178.9 ± 51.2	161.3 ± 38.4	0.660 ^a^	--
**1 month**							
**QOL**		83.59 ± 5.2	82.05 ± 4.8	83.16 ± 3.35	83.5 ± 3.5	0.658	--
**BHQ**		83.0 ± 4.6	83.89 ± 6.16	83.58 ± 4.9	82.67 ± 4.6	0.861	--
**EWL %**		8.05 ± 1.6	8.37 ± 1.8	11.0 ± 1.5	12.3 ± 1.14	**< 0.001**	S*O, S*R G*O, G*R O*R
**3 months**							
**QOL**		84.66 ± 5.03	83.0 ± 4.9	83.8 ± 3.6	84.06 ± 3.8	0.608	--
**BHQ**		83.07 ± 4.2	84.32 ± 6.14	83.68 ± 4.2	82.4 ± 4.8	0.624	--
**EWL %**		26.9 ± 4.04	27.5 ± 3.3	32.7 ± 3.04	35.05 ± 2.4	**< 0.001**	S*O, S*R G*O, G*R
**6 months**							
**QOL**		85.43 ± 4.8	83.95 ± 5.11	84.58 ± 3.7	84.61 ± 4.05	0.666 ^a^	--
**BHQ**		84.16 ± 3.9	84.37 ± 5.3	85.11 ± 3.6	83.17 ± 4.48	0.590 ^a^	--
**Stool analysis**	**Formed**	29 (65.9%)	12 (36.8%)	0 (0%)	0 (0%)	**< 0.001**^b^	--
	**loose**	15 (34.1)	7 (63.2%)	19 (100%)	18 (100%)		
**Stool culture**	**Normal GIT flora**	100 (100%)	100 (100%)	100 (100%)	100 (100%)	--	
**Fecal calprotectin**		64.8 ± 14.6	70.5 ± 10.7	127.4 ± 26.7	124.6 ± 18.8	**< 0.001**^a^	S*O, S*R G*O, G*R
**EWL %**		32.5 ± 3.36	38.8 ± 6.8	62.5 ± 5.47	64.5 ± 3.2	**< 0.001**^a^	S*O, S*R G*O, G*R

Data is presented as mean ± SD or frequency (%), QOL: quality of life, BHQ: Bowel Health Questionnaire, EWL: Early postoperative weight loss, a or *: ANOVA test. b: Chi square test

## Discussion

### Upon Comparing Probiotic vs. Control Groups

Preoperative, patients’ demographic characteristics, QOL and BH scores were similar, while Probiotic users showed significantly higher QOL scores at 1, 3, and 6 months postoperatively. The probiotic group achieved significantly greater EWL across all follow-up points. Improvement in BH scores was notably significant only by 6 months. Lower levels of inflammatory marker (fecal calprotectin) in the probiotic group imply reduced intestinal inflammation. Although “looser stool motions” were more frequently showed in probiotic group, the difference was insignificant.

Along with our results, Yıldız et al., [12] demonstrated that probiotic supplementation enhanced constipation relief and gastrointestinal QoL during early post-LSG period in the probiotic group than control group; additionally, the probiotic group exhibited a higher Gastrointestinal QoL Index, all with *p* < 0.05.

Conversely, Wagner et al., [13] didn’t notice significant effects in neither the gastrointestinal symptoms (GIS) or in small intestine bacterial overgrowth (SIBO) prevalence with probiotics use.

### Upon Comparing Patients According to Type of Surgery

A **EWL**

RYGB patients consistently showed higher EWL than those underwent other surgeries at all postoperative intervals. RYGB and OAGB patients had significantly higher postoperative percent change in EWL than SG or gastric plication.

B **Inflammatory and gastrointestinal effects**

SG was associated with lowest fecal calprotectin levels compared to more anatomically altering procedures like RYGB and OAGB. It also showed the highest frequency of formed stools, possibly. RYGB’s malabsorptive component enhances weight loss but may also increase gut inflammation (higher fecal calprotectin).

III. **QOL and BH**

Despite differences in physiological outcomes, there was no statistically significant variation across surgical types in QOL or BH scores. Hence; according to our study, both QOL and BH score had improved due to usage of probiotic, regardless of type of bariatric surgery, while each of EWL and Fecal calprotectin were affected by both probiotic use and type of bariatric surgery.

In addition to our findings, Fernandes, et al. [14] stated that inflammatory markers found insignificant changes on comparison between groups after supplementation. The decrease in body weight observed in cases undergoing RYGB was 53.8% more significant in the prebiotic group relative to the placebo group, amounting to a − 0.7 kg.

In addition, Kazzi et al. [15] revealed that weight loss was more greater in the probiotic group compared to the placebo group at 6 weeks, but the difference was insignificant at 3 months post-operation. Furthermore, the probiotic group experienced a notable reduction in triglyceride levels over time (*P* = 0.01). The probiotic group exhibited a significant reduction in AST and LDL levels than placebo group (*P* = 0.03).

In contrast, Major et al. [16] found that there were insignificant differences in body weight loss observed between the 2 types of procedures. Progress was noted in the management of obesity-associated conditions. Additionally, the QoL was markedly improved. No distinctions were observed regarding the extent of QoL enhancement among different categories of surgical procedures. No significant differences were identified in the analysis of the impact of body weight loss on QoL enhancement.

A systematic review and meta-analysis by Wang, et al. [17] had revealed which probiotics showed significant effects than the control groups on regulating triglycerides (*p* = 0.006), weight (*p* = 0.05) and vitamin B_12_ (*p* = 0.05). There were insignificant adverse effects associated with the use of probiotics. Another comprehensive systematic review and meta-analysis conducted by Swierz. et al. [8] had indicated that probiotics may have minimal or no impact on %EWL at 6 months, 6 weeks, and 12 months following surgery. They observed a brief enhancement in GI symptoms. There was no significant impact on QoL, and no notable adverse events occurred. Nonetheless, these findings were derived from evidence of considerably low certainty.

Our study had limitations as short follow-up (6 months), no microbiome analysis, heterogeneous surgical groups; it could be due to sample size variations.

## Conclusions

This study indicates that probiotics use following bariatric surgery provides quantifiable advantages in QoL, weight reduction, and gastrointestinal inflammation, irrespective of the surgical procedure performed. Although procedures like RYGB and OAGB yield superior weight loss, they may induce higher gut inflammation. Meanwhile, sleeve gastrectomy appears to be the most gut-friendly in terms of inflammatory markers and bowel consistency, Probiotics may mitigate postoperative intestinal inflammation, common after malabsorptive procedures, Administration of probiotics or digestive enzymes may alleviate symptomatic GI episodes following gastric bypass surgeries and enhance QoL, RYGB offers superior weight loss but higher gut inflammation, SG patients have better gut health but slower weight loss, Probiotics may counteract postoperative dysbiosis.

We recommended that probiotics should be considered as a standard adjunct therapy following bariatric surgery. Long-term studies are necessary to assess the sustainability of benefits and determine the most effective strains and dosages.

## Data Availability

No datasets were generated or analysed during the current study.
